# Navigating the Digital Divide: Exploring the Drivers, Drawbacks, and Prospects of Social Interaction Technologies' Adoption and Usage Among Older Adults During COVID-19

**DOI:** 10.1155/jare/7625097

**Published:** 2025-01-11

**Authors:** Daniel Katey, Sally Chivers

**Affiliations:** ^1^M.A. Program in Interdisciplinary Aging Studies, Trent University, 1600 West Bank Drive, Peterborough K9L 0G2, Ontario, Canada; ^2^Trent Centre for Aging & Society, Trent University, 1600 West Bank Drive, Peterborough K9L 0G2, Ontario, Canada

**Keywords:** COVID-19 pandemic, digital engagement, older adults, social interaction technologies, social isolation

## Abstract

The COVID-19 pandemic underscored the critical role of social interaction technologies (SITs) in mitigating loneliness and social isolation, particularly among older adults. However, challenges such as the digital divide, physical and cognitive declines, and digital literacy gaps persist. This article seeks to explore the drivers, drawbacks, and prospects of SITs' adoption during the pandemic. The paper employs a narrative review approach, using targeted phrases and keywords, including “COVID-19 pandemic and digital engagement,” “digital technologies usage among older adults/people during COVID-19,” and “drivers of digital technologies adoption among older adults/people during COVID-19.” Articles were retrieved through Google Scholar searches conducted between October 2023 and December 2024. In line with key findings, we propose evidence-based recommendations, including user-centered digital communication technology design, the need to balance digital engagement with healthy physical activity, and personalized digital literacy programs, to enhance SITs' accessibility and usability for older adults.

## 1. Background

In the wake of continuous advancements in knowledge and innovation, digital technologies have become deeply entrenched in modern society, reshaping various aspects of our lives, particularly in recent decades [1–3]. This digital revolution involves a broad range of technologies that utilize computers, software, and other electronic devices for information management and communication [4]. Globally, extant research highlights the varying dynamics of digital technology's adoption across different demographic groups, with young adults exhibiting a generally high uptake [5–8].

However, the digital divide remains a critical concern, particularly in its three core dimensions: access, skills, and outcomes [9, 10]. Access refers to the availability of digital tools and infrastructure, skills pertain to the ability to effectively use these tools, and outcomes relate to the benefits derived from digital engagement. For older adults, these levels of the digital divide often intersect with barriers such as socioeconomic status, education, and health challenges, further limiting their ability to fully leverage digital technologies [11].

Meanwhile, the advent of the COVID-19 pandemic led to a proliferation in the adoption of social interaction technologies (SITs), specifically video conferencing technologies (VCTs) such as Zoom, FaceTime, and Microsoft Teams among older adults [12–14]. While this improved adoption reduced some aspects of the digital divide by increasing access to technology, it also highlighted persistent gaps in digital literacy and unequal outcomes, as many older adults struggled to maximize the benefits of these tools [15, 16]. For this article, we consider older adults as a diverse group of people aged 50  years and above with different socioeconomic and racial backgrounds, habits, preferences, and genders. Recognizing the diversity within this age group and to avoid any potential biases associated with using a single term, we also use the terms “older adults” and “older people” interchangeably. This approach reflects the broad spectrum of older adult populations represented in the literature. To further contextualize this paper, we draw on theoretical frameworks such as the diffusion of innovations (DOI) theory by Rogers [17] and the technology acceptance model (TAM) by Davis [18]. The DOI theory provides a lens to understand how new technologies are adopted by individuals, emphasizing factors such as relative advantage, compatibility, complexity, observability, and trialability. TAM complements this by focusing on perceived usefulness and ease of use as determinants of technology adoption. These theories are particularly relevant to understanding the drivers and drawbacks to SIT uptake among older adults during the pandemic.

The literature search was conducted in Google Scholar between October 2023 and December 2024 using specific phrases and keywords, including “SITs' usage among older adults/people,” “COVID-19 pandemic and digital engagement,” “digital technologies usage among older adults/people during COVID-19,” “drivers of digital technologies adoption among older adults/people during COVID-19,” “drawbacks of digital technologies adoption among older adults/people during COVID-19,” and “measures to improve digital technologies adoption among older adults/people.” Leveraging a narrative review approach, this article provides insights into the drivers and drawbacks of the increase in SIT adoption among older adults during the pandemic, as well as proffers pragmatic recommendations to promote inclusivity and accessibility in SITs uptake and usage postpandemic.

## 2. Literature Review

### 2.1. Drivers of SITs' Adoption and Usage Among Older Adults During the Pandemic

According to Ray et al. [13], the COVID-19 pandemic significantly transformed how people engaged and communicated. Isolation due to social distancing and lockdowns prompted some older people to seek alternative means of staying socially connected, leading to a surge in the adoption of popular VCTs platforms such as Zoom, WebEx, WhatsApp Video, Google Meet, FaceTime, Microsoft Teams, and Skype [13, 19]. Thus, video conferencing platforms and social networking services became more accessible and desirable globally, facilitating virtual gatherings and family interactions during the pandemic [20]. In Norway, Badawy et al.'s [19] study conducted among some older adults (aged 87–92 years) and their relatives revealed that digital meetings provided opportunities for older people and their relatives to enjoy each others company by conveying a homey atmosphere.

**Figure 1:**
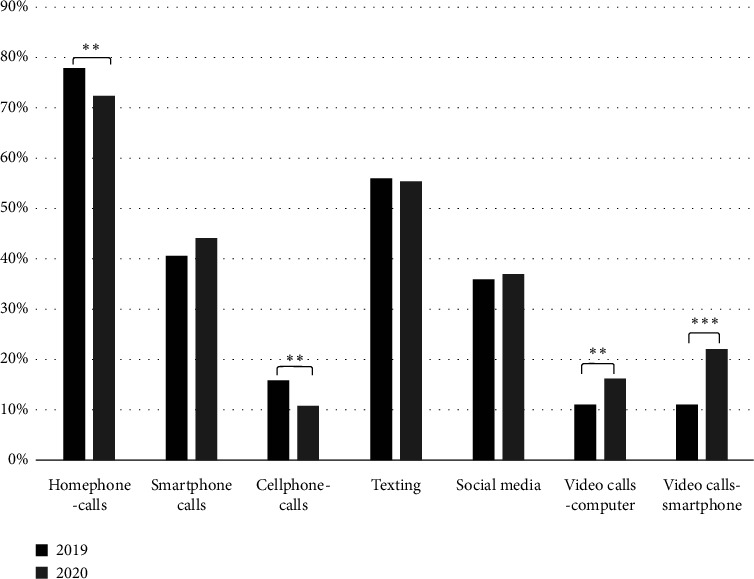
Proportion of participants using different digital communication methods to connect with family and friends, before and during the COVID-19 pandemic (2019 and 2020). Note: ⁣^∗^*p* < 0.05, ⁣^∗∗^*p* < 0.01, and ⁣^∗∗∗^*p* < 0.001. (Source: Sixsmith et al. [21]).

Figure 1 below illustrates the scale-up in the adoption of VCTs during the pandemic. This figure, adapted from Sixsmith et al.'s [21] study, highlights the noticeable increase in the usage of these technologies by older adults during the pandemic, reflecting a global trend in the broader adoption of VCTs in response to COVID-19. 

Furthermore, the COVID-19 pandemic and its corresponding coping mechanisms triggered an increased awareness of available and alternative forms of social engagement [21]. As a result, several institutions and organizations began leveraging SIT platforms, especially for social gatherings and the exchange of goods and services. For instance, senior centers, community groups, and hospital facilities, among others, all embraced SITs' platforms [14]. This move was motivated by the necessity to maintain continuity in services, social engagement, and support networks throughout the lockdowns and social distancing moments [22]. Some social groups and organizations began organizing virtual activities, including religious services, medical appointments, group meetings, and educational sessions. These efforts aimed at addressing the all-round isolation older adults experienced due to restrictions on in-person engagements and interactions. For instance, a recent review article that focused on advances in telemedicine technologies in treating older cardiac patients (people aged 60+ years) revealed that globally, some healthcare facilities for older adults embraced telehealth and remote monitoring technologies (combined with video conferencing with physicians) which allowed for older people to obtain medical treatments without exposing themselves to any potential health risks [23]. These factors, coupled with the continued promotion of social media handles on popular multimedia channels and widely used apps, made older adults more aware of the availability of SITs, which in turn led to increased adoption [21].

Lastly, some older adults were already acquainted with SITs before the pandemic, which facilitated a seamless transition to VCTs usage during COVID-19 [24]. This ability to adapt to technology is consistent with broader studies that emphasize how factors such as physical capabilities and mental well-being can affect older adults' participation in various activities, including the use of digital tools [25]. According to a Pew Research Centre survey, older adults who had past engagement with technology were more likely to accept new digital technologies [26]. Social support systems, including assistance from family, friends, and community organizations also played a pivotal role in encouraging the adoption and use of SITs among older adults [27]. Family members and friends provided crucial guidance and assistance that enabled some older adults to navigate and engage with SIT platforms effectively [24].

### 2.2. Drawbacks of SITs' Adoption and Usage Among Older Adults During the Pandemic

The exclusion of older adults from the design and development of SITs was a major drawback. SITs have the potential to enhance the quality of life for older people; however, there appears to be a notable divide between available SITs and what older adults genuinely need. As Manchester and Jarke [28] noted, the potential of technology (SITs) to address worldwide issues associated with aging populations has mainly gone unrealized. Similarly, negative preconceptions, such as stereotyping older people as feeble and incompetent, often led to their exclusion from technological engineering and development, hindering the creation of SITs that could effectively cater to their unique needs [29, 30]. This exclusion resulted in the development of SITs that were not tailored to the specific needs of older people [28], thereby limiting their ability to fully leverage SITs to minimize social isolation and loneliness during the pandemic, particularly in long-term residential facilities [30].

Also, physical and cognitive limitations presented drawbacks, especially given the above point about the lack of inclusive design. According to Gyasi et al. [31], physical limitations include any situation or condition that inhibits a person's ability to perform given physical functions. Using SITs such as Zoom and Skype involves some form of physicality. As Haase et al. [24] noted, the process of aging could be a risk factor for physical limitations and an important predictor of low SIT adoption and usage. Based on data from the China Longitudinal Survey of Health and Retirement (CHARLS) conducted in 2015 and 2018, Yan et al. [25] analyzed the association between physical function, activities of daily living (ADL), and depressive symptoms in older Chinese adults. The study found that physical functioning declines with age. This predicament, probably, may have inhibited SITs' adoption and usage among this older adult group during the pandemic, as Yan et al. [25] further discovered that numerous daily activities were difficult to perform independently. Moreover, cognitive decline, including memory loss and decreased problem-solving abilities, further complicated the use of SITs among older adults during the pandemic [30]. This created barriers to activities such as remembering passwords, understanding interfaces, and troubleshooting some digital technological issues, thereby reducing SITs' adoption among some older adults. Seifert et al. [30] also noted that older adults residing in long-term care facilities may face challenges in independently using SITs due to physical or cognitive limitations.

Finally, digital illiteracy also emerged as a significant barrier to older adults' engagement with SITs, particularly as the rapid development of SITs created a widening gap [32]. This lack of proficiency was not solely a matter of education or age; it was intricately linked to socioeconomic factors, such as income level, education, and access to resources [32]. Many older adults, especially those from lower socioeconomic backgrounds, may not have the financial means to acquire devices or reliable internet access [32], thereby hindering their ability to learn and utilize these technologies effectively during the pandemic [32, 33]. In Singapore, Ngiam et al. [32] conducted a nonrandomized controlled trial that involved 138 digitally excluded community-dwelling older adults aged ≥ 55 years and of lower socioeconomic status. They discovered that despite the widespread digital adoption in Singapore, older adults, especially those of lower socioeconomic status, still face difficulties in adopting information and communications technology and are typically digitally excluded. In addition, as noted by Yazdani-Darki et al. [34], low levels of formal education often correlate with diminished digital literacy skills, making it more challenging for older individuals to adapt to technological advancements. The prolonged COVID-19 pandemic further exacerbated these challenges, as seen in the evolution of telemedicine in geriatric psychiatry in India [35]. According to Sivakumar et al. [35], in India, while telephone consultations became more common, the effectiveness of these services was hampered by digital illiteracy, sensory issues, and cognitive impairments among some older adults. Thus, addressing digital illiteracy in older adults requires a multifaceted approach that includes improving access to technology and resources, alongside educational initiatives tailored to their specific needs.

## 3. Suggestions for Improving SITs' Adoption and Usage Among Older Adults

 In line with the identified drawbacks, we propose the following recommendations to assist older adults in maximizing the interactive capabilities of SITs and fostering increased integration into the rapidly advancing technological landscape.

### 3.1. Engage Older People in the Design and Development of Digital Technologies

The findings from Chu et al.'s [36] examination of facial image datasets revealed a significant underrepresentation of older adults in the most widely used dataset, with only 0.001% of the photographs featuring older adults. Given that facial recognition technologies are extensively being incorporated into a wider range of SITs including Instagram and Snapchat [37] and societal applications, such as banking, healthcare, and security, a dearth of older adult representation could impede the creation of fair and equitable systems that meet the needs of a wide range of age groups, thus repelling or excluding older people from different situations [37]. Moreover, given that these SITs immensely rely on facial recognition for features such as filters and augmented reality effects, the underrepresentation of older adults in the training dataset may lead to these SITs being less inclusive and engaging for older adults, thereby obstructing their effective participation in digital spaces. Hence, this paper calls for a diversification in the datasets used to train facial recognition algorithms to include an equal representation of all age groups, including older adults. This could be achieved through collaboration with older adults and appropriate institutions to collect high-quality, varied but representative facial data that accurately reflect demographic diversity.

Again, it is also very important to engage older adults in the SIT design process through a user-centered design (UCD) approach. In this iterative design process, technology designers concentrate on the needs and wants of end-users at every stage of the design process [38, 39]. As noted by Jones et al. [40], the experiences and ideas of older adults are indispensable when developing user-friendly interfaces that accommodate their capabilities, choices, and needs. Thus, by using this participatory method, SIT development could meet the distinctive needs and preferences of older users [39].

### 3.2. Balancing Technology-Based Social Interactions With Healthy Physical Activity

Placing undue emphasis solely on increasing SITs utility among older people without comprehensive and guided education on healthy usage dynamics could inadvertently promote sedentary lifestyles among older adults, which is potentially detrimental to their health and overall well-being [41]. All other things equal, the longer the hours older adults spend chatting or communicating with family and friends via SIT platforms, the more socially and emotionally connected they will become. However, there is a critical problem with this immersive digital engagement. Usually, for this connection to build, older adults may find themselves sitting in one place for extended periods or engaging with digital screens for long hours. This sedentary behavior could either trigger pre-existing sedentary lifestyle-related health conditions such as diabetes, obesity, cardiovascular diseases, or musculoskeletal issues or predispose them to the risks of these complications [42]. The World Health Organization (WHO) identifies physical inactivity as a key risk factor for noncommunicable diseases, amplifying the importance of balancing technology-based social interactions with healthy physical activity [43]. Thus, encouraging older adults to embrace SITs for social connectivity is pivotal; however, it should be complemented by extensive education on healthy usage patterns and the integration of physical activity (dancing, running, walking, chair exercises, and yoga) into their daily routines. This could also involve raising awareness of screen time, encouraging breaks, and promoting a mindful approach to SITs usage [1, 44]. In addition, older adults could resort to the use of various software and platforms such as Aaptiv and Fitness Trackers that offer guided workout sessions and fitness tracking, making it easier for them to incorporate regular exercise into their SITs usage routine to improve their cognitive abilities. Finding the equilibrium between technological engagement and physical activity could be essential to ensure the holistic physical and cognitive well-being of older adults.

### 3.3. Addressing Digital Illiteracy

Without any doubt, addressing digital illiteracy among older adults requires a complex strategy that considers both individual and institutional factors. One important approach is to create and implement personalized digital literacy programs for older people [45]. An example could be to provide customized computer lessons for older people [46]. These programs should be open to accommodate different levels of digital literacy and address their specific needs and concerns [47]. This could be supported by available learning resources. For instance, online courses and workshops coupled with user-friendly regulations can give older adults the tools and information they need to develop critical digital skills [46, 47]. However, these resources should be provided in a clear and intelligible manner to encourage effective learning [45]. Furthermore, community-based training initiatives could be implemented to provide an encouraging and familiar atmosphere for older people to learn. Thus, local community centers, libraries, and senior centers can act as hubs for digital literacy courses and training centers, building a feeling of community and shared learning [48]. Another effective technique is to promote intergenerational learning initiatives [49]. In this case, younger people can mentor older people in digital skills, encouraging both skill transfer and the formation of social links between generations. Meanwhile, public awareness initiatives also play an important role in accentuating the necessity of digital literacy for older persons. That is, emphasizing the practical benefits of SITs usage, such as staying connected with family, obtaining knowledge, and engaging in online communities can drive older people to engage in digital learning [34, 50].

## 4. Implications and Recommendations

This article highlights the dynamic factors influencing the adoption of SITs among older adults during the COVID-19 pandemic, offering a basis for actionable insights to foster an inclusive and age-friendly digital environment. To address the digital divide, it is essential to prioritize the inclusion of older adults in the design and development of SITs. This can be achieved by engaging them in usability testing to co-create tools that cater to their specific needs, such as simplifying navigation features and incorporating accessibility elements such as larger text sizes and voice-guided interfaces. In addition, intergenerational learning programs, where younger individuals mentor older adults, can facilitate knowledge transfer and strengthen social ties. Community-driven digital literacy initiatives tailored to the diverse socioeconomic and cultural backgrounds of older adults are equally vital. These programs should provide step-by-step guidance on fundamental skills such as using smartphones and participating in video calls, while also addressing language and cultural issues to enhance accessibility. Furthermore, integrating SITs into broader health and social care systems can enhance their utility. For instance, telehealth platforms designed with simplified login processes and multilingual options can help older adults access medical services more effectively. At the same time, hybrid approaches combining online communications and healthy physical activity can mitigate the risks of sedentary lifestyles often associated with increased technology use. Policymakers should adopt inclusive frameworks that recognize the diverse characteristics of older adults and avoid one-size-fits-all solutions. More so, subsidizing digital devices and internet access for low-income older adults, for example, can address socioeconomic barriers, while equitable representation in policy decisions can ensure their unique needs are met. Finally, sustained research into the long-term effects of SITs on older adults' mental and physical health, social engagement, and overall well-being will provide essential data to inform policies and technological advancements. By addressing these challenges and leveraging the potential of SITs, society can create a sustainable and inclusive digital future that empowers older adults rather than marginalizing them.

However, this article is not without limitations. While it draws on the existing literature to provide valuable insights, it is constrained by the scope of reviewed studies, which may not fully represent the diverse experiences and contexts of older adults across different socioeconomic, cultural, and geographic settings. In addition, the reliance on secondary data limits the ability to directly explore the lived experiences of older adults in adopting and using SITs during the pandemic. Therefore, future research should address these gaps by incorporating empirical studies that capture the perspectives of older adults from diverse backgrounds to ensure a more comprehensive understanding of the factors influencing SIT adoption and usage.

## 5. Conclusion

The incorporation of technology into various elements of modern society has had a substantial impact on how we age, learn, work, communicate, and obtain medical treatments. The need to be socially connected, increasing awareness and accessibility to SIT platforms during the COVID-19 pandemic, and the incidence of prior knowledge and social support services all affected the general adoption of SITs among older adults. Despite these drivers, there are significant drawbacks to SITs adoption, such as the isolation of older adults from the design process, physical and cognitive limitations, and digital illiteracy. Recognizing these challenges, the prospects for SITs' adoption and usage among older adults lie in involving them in digital technology design and development, balancing technology-based social interactions with healthy physical activity, and addressing digital illiteracy through personalized programs, community-based initiatives, and intergenerational learning. In effect, these could go a long way to also enhance the health-seeking behaviors of older adults, especially in developing countries [51].

However, it is crucial to state that in implementing our recommendations to bridge this important digital technological divide, older adults should be considered as a diverse group of people with distinct socioeconomic and cultural backgrounds and should be treated as such. They should never be thought of as a homogeneous group of people, as such erroneous misconceptions would result in a one-size-fits-all approach that could, at best, add insult to injury. This recognition is fundamental, especially for fostering inclusivity and ensuring that the benefits of digital technology development, adoption, and utilization cater to the unique needs of the aging population as well.

## Data Availability

Data sharing is not applicable to this article as no new data were created or analyzed in this study.
